# Is there a causal link between *PTEN* deficient tumors and immunosuppressive tumor microenvironment?

**DOI:** 10.1186/s12967-020-02219-w

**Published:** 2020-01-30

**Authors:** Vildan B. Cetintas, Nizar N. Batada

**Affiliations:** 1 Department of Medical Biology, Faculty of Medicine, Ege University, Izmir, Turkey; 2 Centre for Genomic and Experimental Medicine, MRC Institute of Genetics & Molecular Medicine, University of Edinburgh, Edinburgh, UK

**Keywords:** *PTEN*, Immunosuppressive tumor microenvironment, Immunotherapy resistance, Innate immunity, Interferon, cGAS/STING

## Abstract

The *PTEN* tumor suppressor is the second most commonly inactivated gene across cancer types. While it’s role in PI3K/AKT and DNA damage pathways are clear, increasing evidences suggest that *PTEN* may also promote anti-tumor immunity. *PTEN*-deficient tumors are characterized by (i) reduced levels of cytotoxic T cells, helper T cells and NK cells, (ii) elevated pro-oncogenic inflammatory cytokines like CCL2 and (iii) increased levels of immunosuppressive cells such as MDSCs and Tregs. An intriguing possibility is that link between *PTEN* and anti-tumor immunity is mediated by the interferon signaling pathway. In this review, we summarize the evidences for the mechanistic link between *PTEN* deficiency and immunosuppressive tumor microenvironment and the interferon signaling pathway. We further discuss how the link between these pathways can be exploited for development of personalized immunotherapy for patients with *PTEN* deficient tumors.

## Background: tumour suppressive functions of *PTEN* and prevalence of *PTEN* mutations across cancers

Phosphatase and tensin homolog (*PTEN*) is a dual phosphatase which has both lipid and protein phosphatase activities in cytoplasm and nucleus respectively. Removing one phosphate group from phosphatidylinositol 3,4,5‑trisphosphate (PIP3) inhibits the activity of the phosphoinositide-3-kinase/AKT serine/threonine kinase (PI3K/AKT) pathway to regulate cell proliferation, metabolism, survival, polarity, migration and angiogenesis [1–4]. Moreover, protein phosphatase activity of *PTEN* regulates cell cycle and response to DNA damage in the nucleus [5, 6]. Thus these roles of *PTEN* suggest that its deficiency could lead to increased genome instability by affecting fidelity of the DNA repair pathway called homologous recombination (HR) [7].

**Fig. 1:**
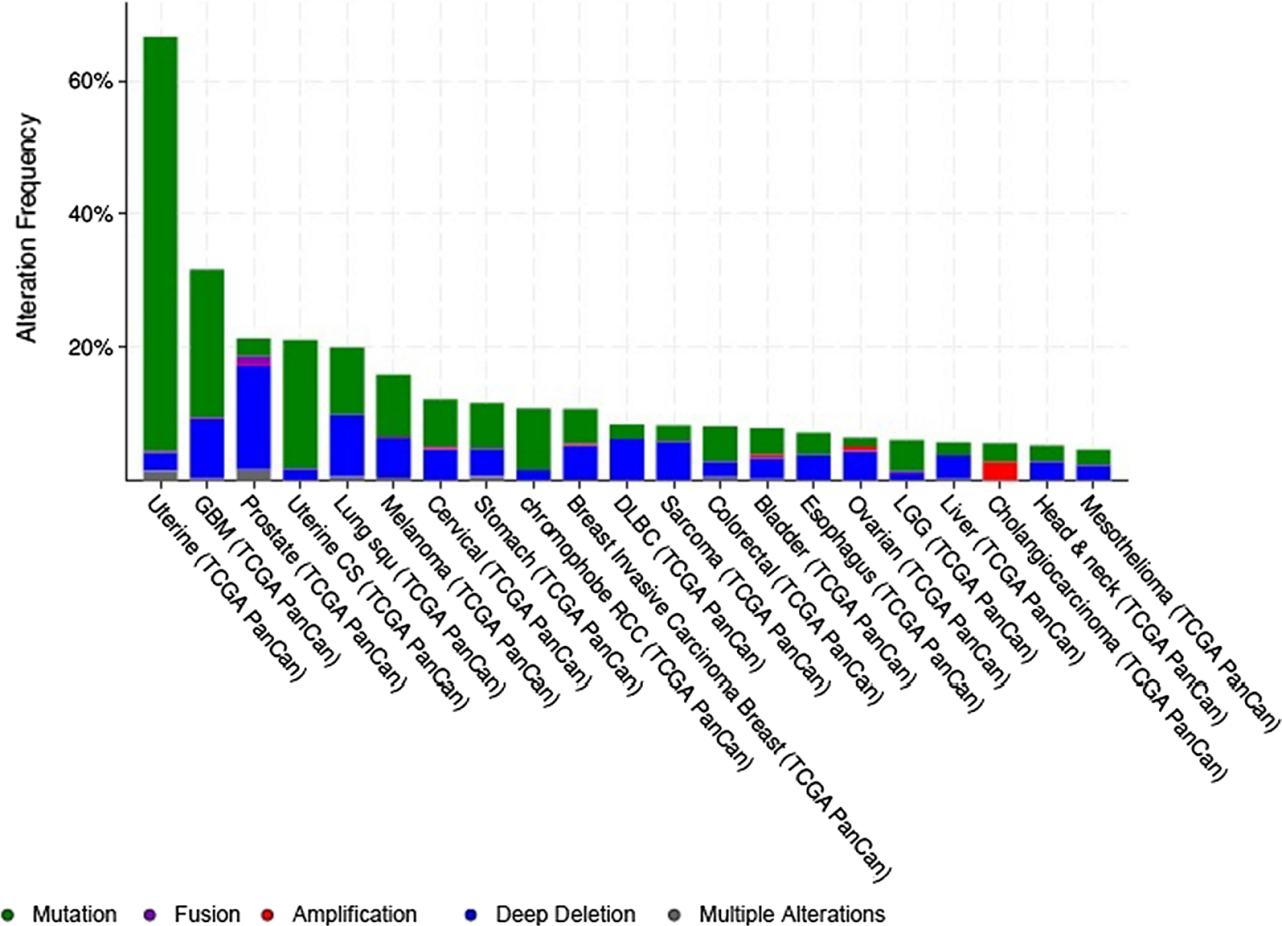
Prevalence of *PTEN* mutations in different cancers. The graph is from cbioportal and has been restricted to pancancer studies

Loss of *PTEN* functions due to genetic aberration or epigenetic silencing has been related to malignant transformation, progression, chemotherapy response and survival in several cancers [8–11]. PI3K pathway alterations were identified in 44% of the 60,991 solid tumors and *PTEN* (9.4%) was the second frequently altered gene after PI3K (13.3%) [12]. Pancancer restricted analyses of different tumors revealed that *PTEN* alterations, mostly mutations and deep deletions, are frequent in uterine, glioblastoma (GBM), prostate, lung and melanoma cancers (Fig. 1).

Deregulation of PI3K signaling pathway resulting from genetic alterations in the *PTEN* have been identified in over 50% of GBMs [13]. *PTEN* mutations are found in 41% of GBM patients and loss of *PTEN* contributed to impeded DNA repair pathway after ionizing radiation [7, 14]. A recent report highlighted that phosphorylation of *PTEN* at tyrosine 240 (pY240) by fibroblast growth factor receptor 2 (FGFR2) mediates radiotherapy (RT) resistance in GBM [15]. Homozygous deletions and missense/truncating mutations of *PTEN* found in 17% of primary prostate cancers [16]. *PTEN* deletion is also associated with intratumor heterogeneity in prostate cancer [17]. In a large cohort of Non-Small Cell Lung Cancer (NSCLC), *PTEN* loss was present in half of the squamous cell carcinoma (SCC) and in one-third of adenocarcinoma (AC), and associated with poorer prognosis [18]. In the TCGA melanoma cohort, somatic *PTEN* alterations were identified in 14% of specimens, consisting of both mutations and focal deletions [19]. Moreover, loss of *PTEN* has been associated with resistance to BRAF inhibitor and decreased overall survival in melanoma [20, 21].

## Evidences for immunosuppressive tumour microenvironment in *PTEN* deficient tumors

Emerging works suggest that *PTEN* might have additional functions in the tumor microenvironment including those affecting tumor growth through modulation of the immune response [30, 31]. Host immune response against tumor cells is a tumor suppressor mechanism which provide a barrier to malignant transformation. *PTEN* signaling influences a broad array of immune cells of both the innate and adaptive compartments (Table 1). Several research groups have reported that *PTEN* loss tumor cells lead up immunosuppressive infrastructure and break down transformation barrier in the tumor microenvironment (TME).

**Table 1:** *PTEN*-mediated immunogenicity in different types of tumors

Tumor	Main evidence	Experimental setup and methods	
Lung cancer [8]	A decrease in *PTEN* expression contributes to cellular unresponsiveness to IFN-γ	Cell lines PC14PE6/AS2 A549	shRNA, plasmid transfection, WB, FC, luciferase reporter assay, intracellular ROS assay
GBM [22]	Tumors had increased levels of B7-H1 protein and tumor-specific T cells lysed human glioma targets expressing PTEN^wt^ more effectively than those expressing PTEN^mutant^	U87MG Cell line and primary cultures	FC, RT-PCR, IHC, WB, NB
Prostate cancer [23]	Cytokines released by *PTEN*-null senescent prostate tumors drive an immunosuppressive TME, Jak2/Stat3 pathway is activated in *PTEN*^pc−/−^ senescent tumors	Mice models Pten^pc+/+^, Pten^pc−/−^ Pten^pc−/−^; Stat3^pc−/−^	MACS, Cytokine array, FACS, CD8^+^ suppression assay, pStat3, WB, IHC, IF, GZMB mRNA, H&E
Melanoma [24]	*PTEN* negatively regulates the expression of immunosuppressive cytokines and PD-L1 by inhibiting the PI3K pathway	Cell lines (*PTEN*-defective vs. *PTEN* expressing pairs)	Expression of the IL-10, IL-6, VEGF, PI3K inhibitor treatment
	Melanoma samples lacking brisk host responses showed a higher tendency to lose *PTEN*	Brisk host response n = 33, without brisk host responses n = 34	IHC (CD3 and *PTEN*)
Melanoma [25]	*PTEN* loss causes resistance to T cell mediated response	Cell line A375 PTEN^silenced^ vs control	shRNA, T cell treatment, Casp3 cleavage assay
		Mice tumor model PTEN^silenced^ vs control	Luciferase expressing T cells treatment, bioluminescence imaging, tumor size, survival
	*PTEN* absent tumor cells have lower CD8^+^ T cell infiltration	Clinical human samples	135 resected tumors, IHC
		TCGA	Lymphocyte activation score, cytolic activity, expressions of LCK, IFNγ, GZMB
	*PTEN* loss promotes resistance to immune infiltration of tumors through the production of inhibitory cytokines	Mice xenografts model PTEN^silenced^ vs control	Chemokines and cytokines by Luminex assay
		Clinical human samples	IHC confirmed increased VEGF in regions with *PTEN* loss
Sarcoma [26]	*PTEN* loss is associated with induction of an immunosuppressive microenvironment and resistance to PD-1 blockade	Primary tumor, treatment-resistant metastatic tumor and germline tissue from a clinical case	IHC, RNA-seq and WES
	Tumors with biallelic *PTEN* loss had significantly lower levels of mRNA expression of PDCD1, CD8A, IFNG, PRF1, and GZMA compared to PTEN^wt^ tumors	TCGA	Mutation, copy number, RNA-seq data from 241 untreated primary sarcomas
Prostate cancer [27]	*PTEN* loss leads to upregulated inflammatory and cytokine–cytokine receptor signaling.	*PTEN* null murine models Cell lines	FACS, IHC, Q-RT-PCR, T cell suppression assay, laser capture and microarray
	Pro-inflammatory cytokines produced by *PTEN* null prostate are the major causes of MDSC expansion		
Lymphoma [11]	Low *PTEN* mRNA expression is associated with down-regulation of a group of genes involved in immune responses and B-cell development/differentiation and poorer survival	478 cases (training cohort) 269 cases (validation cohort)	IHC, FISH, Gene sequencing and expression array
GBM [28]	*PTEN* mutations associated with immuno suppressive expression signatures in ICIs non-responders	66 patients treated with PD-1 inhibitors profiled across a variety of timepoints, collecting DNA, RNA, tissue imaging	WES, qmIF, lymphocyte clonality analysis, RNA seq
Prostate cancer [29]	FoxP3^+^ Tregs were significantly increased in *PTEN* deficient PCa, *PTEN* deficiency is linked to an immunosuppressive state in PCa with distinct changes in the frequency of immune cell types in tumors from different metastatic sites	741 primary and 96 metastatic tumors, 94 radical prostatectomy specimens for IH validation	in silico analysis and IH validation for IDO1 and PDL1

*FACS* fluorescence activated cell sorting, *FC* flow cytometry, *FISH* fluorescein in situ hybridization, *GBM* glioblastoma, *GZMB* granzyme B, *H&E* hematoxylin and eosin, *IF* immunofluorescence, *IHC* immunohistochemistry, *IL* Interleukin, *LCK* lymphocyte cell-specific protein-tyrosine kinase, *MACS* magnetic-activated cell sorting, *MDSC* myeloid-derived suppressor cell, *MHC* major histocompatibility complex, *NB* Northern blot, *PI3K* phosphoinositide 3-kinase, *qmIF* quantitative multiplex immunofluorescence analysis, *RT-PCR* reverse transcription-polymerase chain reaction, *ROS* reactive oxygen species, *shRNA* short hairpin RNA, *TCGA* the cancer genome atlas, *TME* tumor microenvironment, *VEGF* vascular endothelial growth factor, *WB* Western blot, *WES* whole exome sequencing

The first evidence of *PTEN* and immune homeostasis was reported that germline deletion of *PTEN* manifests autoimmune disorders [32]. Type II Interferon (IFN)-γ acts on tumor cells, enhancing their recognition by CD8^+^ T cells as well as by CD4^+^ T cells, and unveiling a key role in the promotion of tumor immunogenicity [33]. Therefore, major efforts have been made for the development and establishment of combined clinical therapeutic applications [34–37]. Src homology-2 domain-containing phosphatase-2 (SHP2), an oncogenic phosphatase, inhibits type II IFN-γ signaling. It was demonstrated that lung adenocarcinoma cells, which express low levels of *PTEN*, are unresponsive to IFN-γ and restoring *PTEN* expression reverses cellular unresponsive to IFN-γ [8]. *PTEN* loss also caused immune escape from IFN-γ-mediated cell proliferation inhibition and cytotoxicity in lung adenocarcinoma cells [8].

Loss of *PTEN* increased the level of PD-L1 (B7-H1) expression through regulation of translation and it is associated with immunotherapy resistance in patients with GBM [22]. *PTEN*-null prostate senescent tumors can promote growth of adjacent non-senescent tumor cells and cause chemoresistance through the senescence associated secretory phenotype (SASP) associated mechanism [23]. These tumors are characterized by increased levels of several cytokines, strongly infiltrated by granulocytic myeloid-derived suppressor cells (MDSCs), in absence of CD4^+^, CD8^+^, and natural killer (NK) infiltrates. Moreover, tumor-infiltrating MDSC cells suppressed the proliferation of CD8^+^ T cells and inhibited their cytotoxic functions [23].

*PTEN* has been reported as a molecular biomarker to predict brisk host response in melanoma cells [24]. According to this, testing *PTEN* will be useful to identify and recruit melanoma patients that might respond better to immunotherapies [24]. Peng et al. [25] remarked *PTEN* loss as a resistance marker to T cell-mediated antitumor immune responses in melanoma. *PTEN* loss was associated with decreased numbers and impaired function of tumor-infiltrating T cells and inferior outcomes with anti-PD-1 treatment. Loss of *PTEN* in melanomas promoted resistance to immune infiltration of tumors through the production of inhibitory cytokines, C-C motif chemokine ligand 2 (CCL2) and vascular endothelial growth factor A (VEGF) which contributes to the immunosuppressive tumor microenvironment by recruiting suppressive immune cells [25]. Peng’s study delineated the influence of an oncogenic pathway on antitumor immunity and response to immunotherapy [38].

*PTEN*-mediated mechanism of immune resistance to anti-PD-1 therapy was also confirmed in a case report from a chemotherapy-naïve patient with rapidly-progressive metastatic uterine leiomyosarcoma who experienced complete tumor remission for > 2 years on anti-PD-1 monotherapy [26]. VEGFA expression increased and PD-1^+^ T cell infiltration reduced in the treatment-resistant mesenchymal tumor with biallelic *PTEN* loss [26]. It was also suggested that *PTEN* loss causes prostate cancer initiation and progression by upregulation of inflammatory and cytokine–cytokine receptor signaling pathways and these associate with marked chronic and extensive MDSCs immune cell infiltration [27]. Comparative analysis of prostate cancer models showed that the diverse genetics of prostate cancer with *PTEN* loss can directly determine the differential infiltration and composition of immune cells in the TME [39]. Major tumor drivers can activate proinflammatory and immunosuppressive programs and at gene-specific intrinsic pathways are at the core of diverse protumoral immune-cell recruitment and infiltration [39].

Diffuse large B-cell lymphoma (DLBCL) patients with low *PTEN* mRNA levels had significantly poorer overall survival and progression-free survival [11]. Distinct gene expression signatures were identified for low *PTEN* mRNA expression compared with *PTEN* mRNA^not low^. The spectrum of *PTEN*-mRNA^low^ genes showed downregulation of genes involved in immune responses, B-cell receptor (BCR) signaling, gene expression and metabolism [11].

Overexpression of *PTEN* induced a large number of common differentially expressed genes in the *PTEN*-null GBM cell line [40]. Several cytokines such as interleukin (IL)-6, IL-8, and IL16 that are highly expressed in GBM were downregulated by *PTEN* overexpression [40]. It was suggested that downregulation of these proto-oncogenic inflammatory cytokines by *PTEN* affect not only the GBM cells but also the crosstalk between tumor cells and the microenvironment, both of which are contributing factors in suppressing tumor growth. In a recent study, somatic *PTEN* mutations were associated with resistance to immune checkpoint inhibitors (ICIs) by altering immunosuppressive environments in GBM [28]. *PTEN* was significantly more frequently mutated in the non-responsive tumors than in the responsive ones and immunosuppressive signature of GBM was most associated with the CD44^+^ tumor sub-population of the *PTEN*-mutated case [28].

In a metastatic melanoma cohort, higher burden of copy number loss was observed in non-responders compared to responders on cytotoxic T-lymphocyte associated protein 4 (CTLA-4) blockade [41]. *PTEN* was identified as one of the tumor suppressor genes with recurrent copy number loss from patients with high burden of copy number loss in this study. Copy number loss burden and down-regulation of immune related gene expression was correlated so it was suggested that there may be gene expression sequelae of extensive copy number loss, including *PTEN* loss [41].

**Fig. 2:**
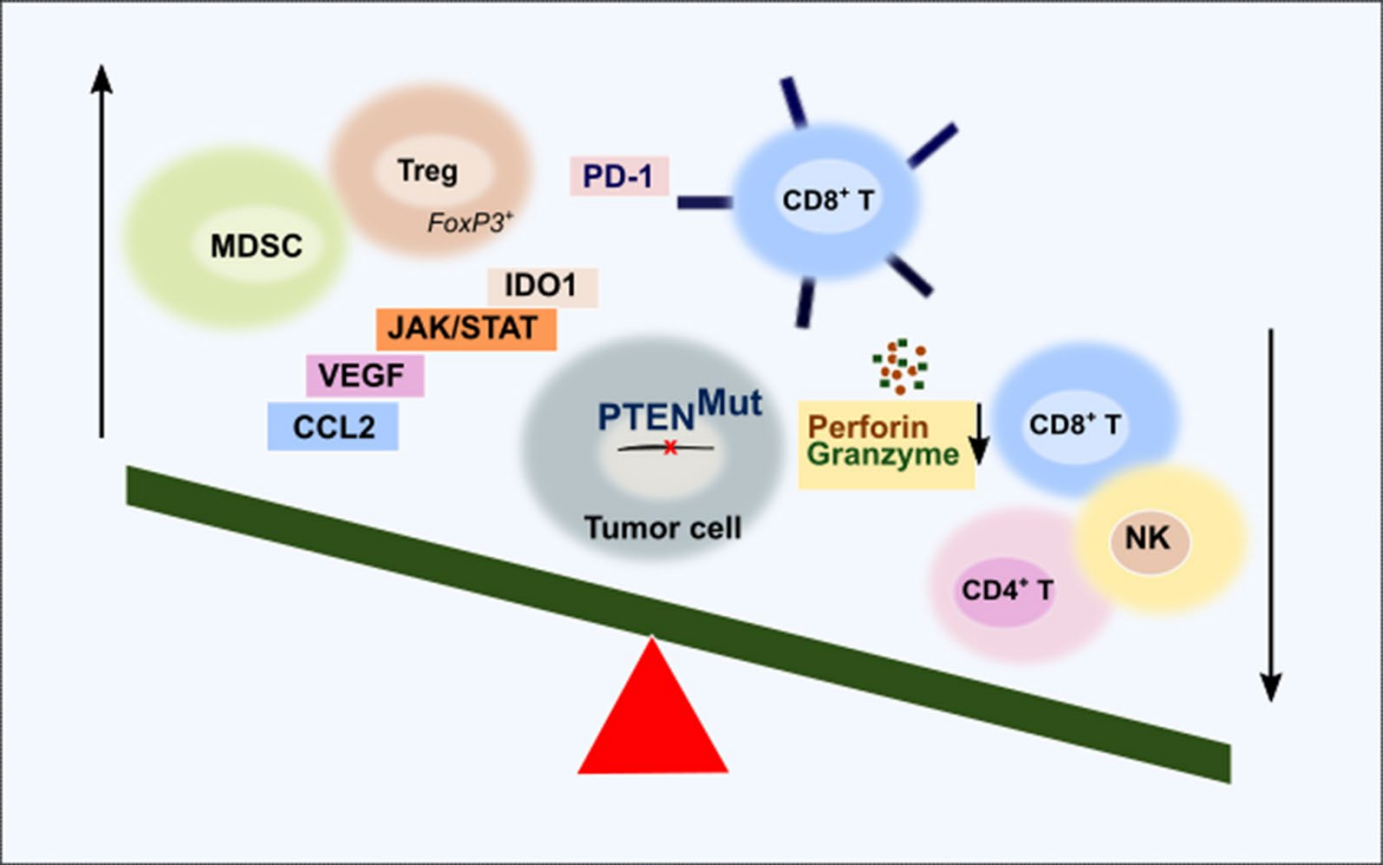
Immunosuppressive characteristics of *PTEN* mutant tumors. *PTEN*-deficient tumors are infiltrated by MDSCs [27] and Tregs [29]. JAK/STAT pathway is activated [23], IDO1 protein [29], PD-1 receptors [22] and inhibitory cytokines [23, 25, 27] are upregulated. CD4^+^, CD8^+^ and NK cells exhibited reduced infiltration [23, 25]. Cytotoxic T lymphocytes have reduced lysing activities depending on the granzyme and perforin depletion [25, 26]

*PTEN* in colonic smooth muscle cell could modulate cytokines/chemokines production to affect the immune cells recruitment to mucosa of colon [42]. Pancreatic ductal adenocarcinoma (PDAC) genome has frequent deletion of the *PTEN* as well as loss of expression in primary tumor specimens. The mouse PDAC driven by oncogenic Kras and *PTEN* loss promotes marked nuclear factor kappa B (NF-κB) activation and its cytokine network, with accompanying robust stromal activation and immune cell infiltration [43]. Recently, *PTEN* deficiency has been linked to an immunosuppressive state in prostate cancer with distinct changes in the frequency of immune cell types in tumors from different metastatic sites [29]. Forkhead box P3^+^ (FoxP3^+^) regulatory T cells (Treg) cells and overexpression of indoleamine 2,3-dioxygenase 1 (IDO1) protein were reported as the source of immunosuppression [29] (Fig. 2).

## Possible mechanisms that link *PTEN* deficiency with immunosuppressive tumour microenvironment

So far *PTEN* deficiency has been linked to promoting tumors *indirectly* through dysregulation of PI3K/AKT and DNA damage. However, mounting evidences suggest that *PTEN* loss can also *directly* contribute to immunosuppression of the tumor microenvironment. More specifically, *PTEN*’s deficiency can lead to immunosuppressive tumor microenvironment due to inability of *PTEN*-deficient cells to activate the interferon signaling pathway.

Interferons (IFNs), type I, II and III, are pleiotropic immunomodulatory class II cytokines that were discovered as the factors underlying viral interference [44–47]. During the past decades, the precise role of IFNs in the natural immune response to cancer has begun to be understood [48–50]. Immunomodulatory effects of type I IFNs can modify the local immune suppressive tumor microenvironment acting on both innate and adaptive immune components [51, 52]. IFN signaling has been show to promote immunity in multiple ways as follows: (a) stimulating the maturation of dendritic cells (DCs) from monocytes in the presence of IFN-α, enhancing their capacity to process and present dead cell associated antigens, and promoting their migration towards lymph nodes [53], (b) generation of cytotoxic T lymphocytes (CTLs), boosting their immune effector functions by increasing the expression of perforin 1 and granzyme B, and promoting the survival of memory CTLs [54–56], (c) activation of NK cells, and also preventing the elimination of antigen-activated CD8^+^ CTLs by NK cells [57–59], (d) inactivation of the suppressive function of Tregs through a pathway that involves the activation of phosphodiesterase-4 and the consequent depletion of cyclic-AMP (cAMP) [60], and (e) stimulating the release of pro-inflammatory cytokines (such as IL-1β and IL-18) by macrophages [61].

Cytosolic DNA sensing pathway (cGAS-STING) is one of the strong inducer of type I IFNs and other inflammatory cytokines in immune and non-immune cells [62, 63]. This strong inflammatory signaling recruits cytotoxic leucocytes and prime T-cell responses, leading to whole tumor regression [64]. *PTEN* controls the import of interferon regulatory factor 3 (IRF3), a master transcription factor responsible for IFN production, into the nucleus [65, 66]. Thus, deficiency in *PTEN* can account for the inactivation of several cellular defense pathways simultaneously, which renders cells unable to use interferon production to defend themselves [67].

IFNs can be activated through intra- and extra tumor mechanisms to induce immune cells to effectively eliminate tumors and overcome the immunosuppressive tumor microenvironment.

### i. Intra-tumor mechanisms

**Fig. 3:**
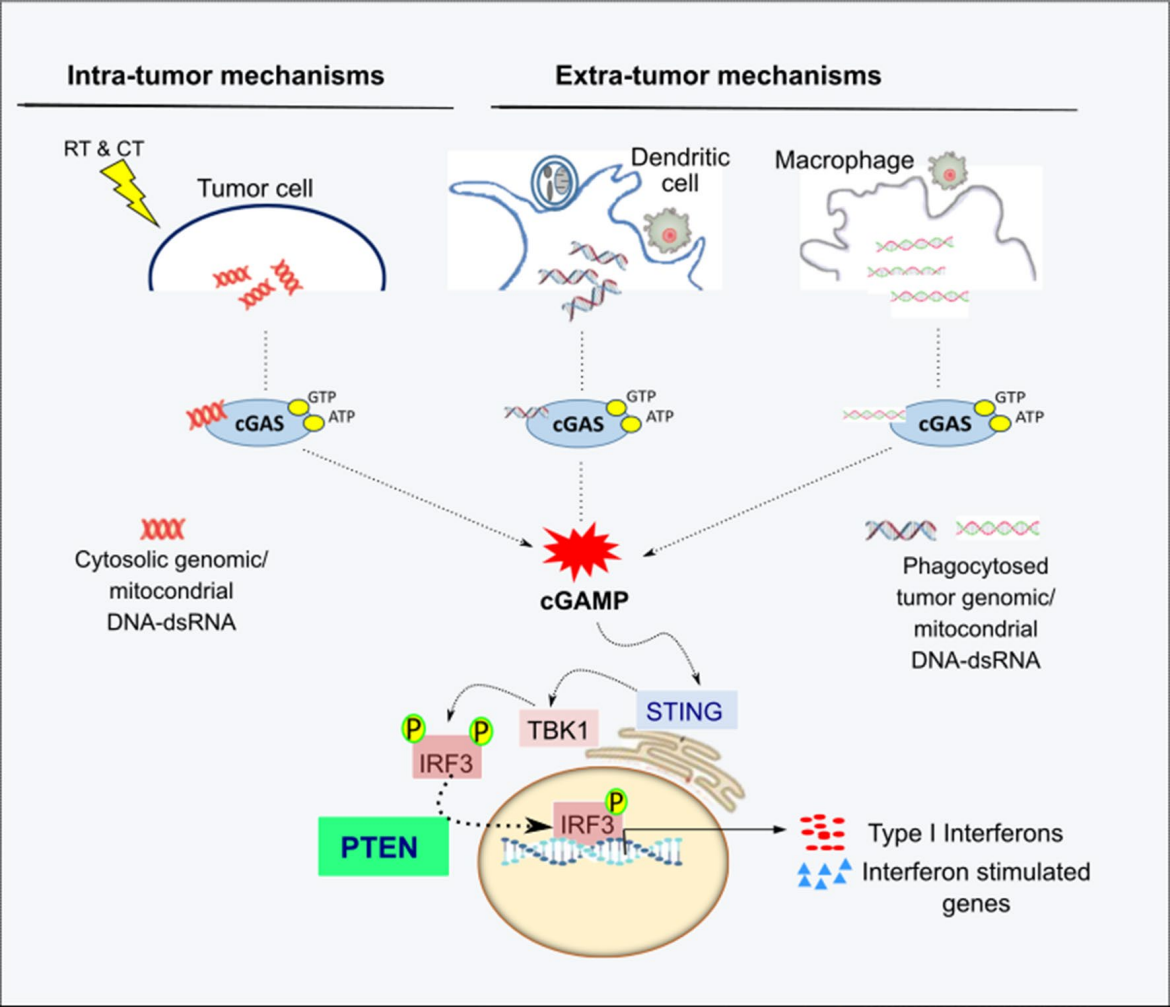
The potential mechanism of *PTEN* in type I interferon mediated immunogenicity. Cytosolic DNA sensing can be activated by intra or extra tumor mechanisms. In the tumor cells, cytosolic genomic or mitochondrial DNA binds to cGAS to trigger the production of cGAMP. cGAMP activates STING and then transcription factors IRF3 and NF-KB. *PTEN* dephosphorylates IRF3 and activates its import to the nucleus and starts the transcription of type I IFN and interferon stimulated genes (ISGs). In the macrophages and DCs, phagocytosed tumors genomic or mitochondrial DNA also activates cGAS/STING pathway

In the tumor cells, cytosolic DNA sensing pathway is induced by various forms of genotoxic stress; DNA damaging drugs, ionizing radiation, oxidative stress, replicative stress, oncogenic signaling, and chromosomal missegregation [68]. Nuclear DNA damage generates cytoplasmic DNA by missegregated chromosomes in subsequent cell divisions which will form micronuclei [64]. Cytoplasmic DNA binds to cGAS in a sequence independent manner and trigger the production of cGAMP which acts as a second messenger to activate stimulator of interferon gene (STING) on the endoplasmic reticulum surface [69]. STING then activates transcription factors IRF3 and NF-KB through the protein phosphatase activity of *PTEN* to elicit the IFNs and cytokines (Fig. 3) [63]. Mitochondria has extensive overlapping transcriptional units and stress associated perturbation of transcript processing can lead to the accumulation of dsRNAs leading to MDA5/RIG1 mediated activation of IFN signaling [70].

### ii. Extra-tumor mechanisms

Necrotic or apoptotic tumor cells can release free or vesicle-protected DNA which likely be phagocytosed by macrophages and DCs. Tumor-derived nucleic acids are taken up by host antigen presenting cells (APCs), translocate into cytosol, trigger the cGAS/STING pathway and contribute to the antitumor immune responses [71, 72]. Phagocytosed tumor derived mtDNA was also recognized by cGAS in the DC cytosol, contributing to type I IFN production and antitumor adaptive immunity [73]. Intratumoral injection of cGAMP transiently induced migration of macrophages into tumor site in a STING-dependent manner and these cells exhibit phagocytosis and tumor necrosis factor α (TNFα) production [74].

## Exploiting immunotherapies in *PTEN* deficient cancers

*PTEN* loss cause immunosuppressive microenvironment through; disruption of lymphocyte infiltration dynamics, upregulation of inhibitory cytokines, decreasing the lysing activities of cytotoxic T lymphocytes depending on the granzyme and perforin depletion. Cancer types such as GBM and prostate, in which *PTEN*-deficiency is common, have low to moderate level of mutations so they would not have many neoantigens which correlates with resistance to ICIs. Thus, determining and considering of *PTEN* status and selection of patients to recovery of the immunogenicity before the immunotherapy may increase the success of immunotherapy.

### PTEN deficient tumors do not necessarily have a better response to immune checkpoint inhibitors

The effects of the *PTEN* loss on the PD-L1 expression have been studied in several cancers. Some clinical data indicates that loss of *PTEN* is associated with elevated PD-L1 levels. However, some studies do not support the role of *PTEN* in regulation of PD-L1.

*PTEN* loss did not show correlation with PD-L1 expression in prostate and breast cancers, high grade neuroendocrine carcinoma of the lung, pulmonary squamous cell, adenocarcinoma, pulmonary sarcomatoid and endometrial carcinoma [89–94]. Although PD-L1 expression was significantly correlated with tumor grade with all PD-L1^+^ cases, mutations of *PTEN* did not correlated with increased intratumoral expression of either PD1^+^TIL or PD-L1 in GBM [75]. Expression of PD-L1 was investigated in a panel of 51 melanoma cell lines and similarly no association was found between the level of PD-L1 expression and mutations in *PTEN* [76] which was confirmed by Peng et al. [25]. TCGA data showed that basal-like tumors, the majority of which were triple-negative breast cancers (TNBCs) showed *PTEN* mutation or loss in 35% of tumors, which also correlated with PI3K pathway activation [77]. However, homozygote deletion of *PTEN* or activating mutation in phosphatidylinositol-4,5-bisphosphate 3-kinase catalytic subunit alpha (PIK3CA) was not associated with increased expression of PD-L1 in TNBCs [78]. In the diffuse large B-cell lymphoma, loss of cytoplasmic *PTEN* was associated with TP53 mutations higher *PTEN*-targeting microRNA expression and lower mean level of PD-L1 expression whereas *PTEN* deletion/mutation and expression of p-AKT, PI3K, or nucleoplasmic-*PTEN* had no association with PD-L1 expression [11].

Low *PTEN* mRNA expression was associated with down-regulation of a group of genes involved in immune responses and B-cell development/differentiation, and poorer survival in DLBCL independent of AKT activation [11]. PD-L1 expression levels and *PTEN* were significantly associated with glandular component of adenosquamous cell carcinoma, whereas there were no associations for the adenocarcinoma and squamous components of lung squamous cell carcinoma [79]. Biallelic inactivation of serine/threonine kinase 11 (Lkb1) and *PTEN* in the mouse lung activated the Akt and mTor pathways and lead to squamous phenotype with elevated PD-L1 expression [80]. *PTEN* loss with increased PD-L1 was reported by Parsa and colleagues in GBM cell lines and they also suggested the involvement of the PI3K pathway [22]. It was confirmed in the breast and prostate cancer cell lines that *PTEN* loss significantly associated to increased PD-L1 expression levels [81]. Likewise, *PTEN* loss led to upregulation of the PD-L1 expression in TNBC and colorectal cancers [82, 83].

The disagreement in the results of these studies may be due to the differences in signaling context of cancers or in association with other genes highlighting that multiple mechanisms may be involved in PD-L1 regulation in tumors. Further clinical studies applying precision genomics and well annotated clinical samples are needed to define the role of *PTEN* on the PD-L1 expression.

### Activating the IFN pathway for treatment of PTEN deficient tumors

Since macrophage polarization is major mechanism of escape from immune control of cancer growth, targeting of tumor-associated macrophages (TAMs) as a promising therapeutic strategy for cancer [84, 85]. Therapies such as anti-CSF1R and anti-CD47 that deplete to M2 myeloid cells are undergoing clinical trials. After CSF1R inhibition, TAMs lose M2 polarization and show enhanced phagocytosis, providing a molecular corollary for their impaired tumor-promoting functions [86]. PLX3397, an inhibitor of CSF1R, blocked glioma progression, markedly suppressed tumor cell proliferation and reduced tumor grade in proneural glioma mouse model [87]. After anti-CD47 blockade, tumor-associated microglia was able to effectively phagocytize tumor cells [88]. However, interfering with these receptors can have severe side effects such as toxicity or autoimmunity as they are also present in non-tumor compartment as well.

An alternative approach that may have benefit is exploiting the IFN signaling pathway [89]. RT increased intratumoral production of IFNβ and enhanced the cross-priming capacity of tumor infiltrating DC from wild type mice but not type I IFN receptor deficient mice [90]. Delivery of exogenous IFNβ into the tumor tissue in the absence of RT is also sufficient to selectively expand antigen-specific T cells leading to complete tumor regression [90]. IFN-β/Temozolomide (TMZ) combination therapy provided suppression of further tumor growth and prolonged survival were achieved in the majority of the malignant gliomas refractory to TMZ [91].

STING was required for type I IFN-dependent antitumor effects of radiation and radiation-induced adaptive immune responses [71]. Combination treatment with the cancer vaccine STINGVAX, a STING agonists, and immune checkpoint inhibitors produces synergistic antitumor effects, which indicates that the cGAS–STING pathway is important for the sensing of tumors by the innate immune system and has a critical role in intrinsic antitumor immunity [92, 93]. STING significantly contributed to antiglioma immunity via enhancement of type I IFN signaling in the tumor microenvironment and suggested a potential use of STING agonists for the development of effective immunotherapy [94]. However, we do not know yet how *PTEN* mutations affect cGAS/STING activity and IFN release. Therefore, further studies are needed to better understand *PTEN*’s role in modulating interferon pathway and cytokine signaling to the tumor microenvironment to develop effective immunotherapy targets.

After the new function for the *PTEN* in regulating IFN responses to viral infection was reported, it was speculated that disruption of *PTEN* function might define the opportunity for viruses to kill cancer [67]. Oncoviral immunotherapies are rising as a novel therapeutic class which has a markedly lower rate of serious adverse effects and greater specificity to target tumor cells [95]. *PTEN* expression by an oncolytic herpesvirus lysed the bulk tumor mass while creating an ATP-rich immune stimulating microenvironment during infection and decreased PD-L1 expression on the surface of tumor cells after treatment, in a murine model of breast cancer with brain metastases and intracranial human GBM tumors in nude mice [96]. Reconstitution of *PTEN* expression during oncolysis can enhance the antitumor immunity and overcome tumor immune escape. However, more work is needed on safety and efficacy evaluation of arming oncolytic herpesviruses with *PTEN*.

## Conclusion

Several functions ensure PTEN the master regulator of physiological processes such as cell metabolism, motility, polarity, genome integrity, proliferation and viability. This review highlights the effects of *PTEN* deficiency on immunosuppressive TME and exploiting immunotherapies in *PTEN* deficient tumors (Table 2). *PTEN* loss can directly determine the differential infiltration and composition of immune cells in the TME and response to immunotherapy. In this case how could immunotherapy apply to *PTEN* deficient tumors? Considering of *PTEN* status and selection of patients to recovery of the immunogenicity before the immunotherapy may increase the success of immunotherapy. *PTEN*’s role in the interferon signaling suggests that tumors from tissues such as brain, breast, ovarian and prostate which poorly respond to existing checkpoint inhibitors, may benefit from activating interferon signaling particularly in *PTEN* deficient tumors where this pathway is expected to have been suppressed.

**Table 2:** Summary of the facts that link PTEN loss in cancer to immunosuppression

Function	Facts
PTEN’s role in tumor suppression	* PTEN deficiency is observed in nearly 40% of glioblastoma [14] * PTEN contributes to repair of DNA damage via the homologous recombination pathway [7] * PTEN deficiency is associated with malignant transformation, chemotherapy resistance and reduced survival [8–11]
Tumors with PTEN deficiency have dysregulated infiltration of immune cells	* High levels of MDSCs [27] and Tregs [29] in the TME of PTEN deficient tumors * Reduced infiltration of CD4^+^, CD8^+^ and NK cells [23, 25] and reduced lysing activities of cytotoxic T lymphocytes depending on the granzyme and perforin depletion [25, 26]
PTEN’s role in type 1 IFN pathway	* Type 1 IFN pathway promotes anti-tumor immunity [49] * PTEN is required for activation of STING mediated induction of interferon alpha/beta gene expression [67]
Potential ways in which PTEN deficient tumors can be targeted by immunotherapies	* Activation of interferon alpha/beta signaling [89, 91] * Engineered PTENα expressing oncolytic viruses can enhance the development of antitumor immunity [96]

## Data Availability

The datasets generated during and/or analysed during the current study are available in the Genomic Data Commons Data Portal repository, https://portal.gdc.cancer.gov/.

## Abbreviations

AC: adenocarcinoma
AKT: AKT serine/threonine kinase
APCs: antigen presenting cells
BCR: B cell receptor
cAMP: cyclic-AMP
CCL2: C-C motif chemokine ligand-2
CTLs: cytotoxic T lymphocytes
CTLA-4: cytotoxic T-lymphocyte associated protein 4
DCs: dendritic cells
DLBCL: diffuse large B-cell lymphoma
FGFR2: fibroblast growth factor receptor 2
FoxP3: Forkhead box P3
GBM: glioblastoma
HR: homologous recombination
ICIs: immune checkpoint inhibitors
IDO1: indoleamine 2,3-dioxygenase 1
IFN: interferon
IL: interleukin
IRF3: interferon regulatory factor 3
ISGs: interferon stimulated genes
MDSCs: myeloid-derived suppressor cells
NF-κB: nuclear factor kappa-B
NK: natural killer
PDAC: pancreatic ductal adenocarcinoma
PIK3CA: phosphatidylinositol-4,5-bisphosphate 3-kinase catalytic subunit alpha
PI3K: phosphoinositide-3-kinase
PIK3: phosphatidyl inositol 3-kinase
*PTEN*: phosphatase and tensin homolog
PIP3: phosphatidylinositol 3,4,5‑trisphosphate
RT: radiotherapy
SASP: senescence associated secretory phenotype
SCC: squamous cell carcinoma
SHP2: Src homology-2 domain-containing phosphatase-2
STING: stimulator of interferon gene
NSCLC: non-small cell lung cancer
TAMs: tumor-associated macrophages
TME: tumor microenvironment
TMZ: temozolomide
TNBCs: triple-negative breast cancers
TNFα: tumor necrosis factor-α
Tregs: regulatory T cells
VEGF: vascular endothelial growth factor-A
